# Intravenous furosemide in decompensated heart failure: do not protocolize dosing but the desired effect!

**DOI:** 10.1186/s13054-014-0709-4

**Published:** 2014-12-22

**Authors:** Harm-Jan S de Grooth, Armand R Girbes, Pieter R Tuinman

**Affiliations:** Department of Intensive Care, VU University Medical Center, P.O. Box 7057, 1007 MB Amsterdam, The Netherlands

Palazzuoli and colleagues report on a study comparing continuous versus bolus infusion of furosemide in acute decompensated heart failure [1]. Patients in the continuous infusion group had greater urine output, decreased renal function and increased 6-month mortality.

We feel that, due to the study design, the conclusions drawn are difficult to translate into clinical practice. The decisive advantage of continuous over bolus infusion is the ability to titrate to effect in a gradual and precise manner. This advantage is completely negated when large-step (that is, doubling) dose increases are rigidly protocolized, as was the case in this study. When the advantages of a method are lost, only its comparative harms remain: continuous infusion may have a stronger diuretic effect than bolus infusion (milligram for milligram) [2] and the continuous infusion group in this study seemed overtreated, resulting in renal dysfunction that is associated with poor outcomes [3].

Future research should focus on the optimal method to attain the clinical goal of inducing sufficient but not excessive diuresis. Comparing outcomes on a protocolized-dose basis is straightforward but cannot elucidate which method is best in practice. Indeed, the authors write that a study utilizing a more tailored dose could further clarify this subject. We suggest that a truly relevant comparison can only be made when treatment in both groups is aimed at individual targets. This would require a large trial with patients optimally treated using either of the two infusion methods, while dosing is completely determined by preset clinically relevant endpoints.

## Author’s response

Alberto Palazzuoli and Gaetano Ruocco

We thank de Grooth and colleagues for their observation. We partially agree with their statements for at least three principal reasons. First, the impact of worsening renal function (WRF) was recently questioned and data from the ESCAPE trial [4], as well as other recent studies, revealed that baseline renal dysfunction but not WRF is related to poor outcome. Secondly, the temporal trend of WRF appears to have a better prognostic significance; in fact, transient or persistent WRF seems to show different impact in terms of outcome [5]. In our study we only measured renal function changes during hospitalization and not after discharge. Finally, in our analysis we did not evaluate signs of congestion, so the relation among overdiuresis/decongestion and WRF/adverse events could only be supposed [1]. However, worse diuretic response is associated with more advanced heart failure, renal dysfunction and poor outcome [6]. It is therefore hard to establish whether impaired renal function is the ‘egg’ or the ‘chicken’.

Instead we agree with the advice that a fixed diuretic administration is far from perfect and its dosing and modality infusion should be adjusted for each patient on the basis of clinical signs, laboratory biomarkers (renal function, hemoconcentration, plasma osmolarity) and kidney imaging. Overall, a guideline standardized method does not currently exist, and therefore all previous studies employed fixed protocols. We indeed propose a more consistent clinical approach measuring daily urine output and weight loss.

Better assessment of systemic congestion considering perfusion, neuroendocrine activation and fluid redistribution could lead to a better understanding of diuretic therapy administration.

## Abbreviation

WRF: Worsening renal function
